# The complete mitochondrial genome of *Epeorus herklotsi* (Ephemeroptera: Heptageniidae) from Longquan, Zhejiang, China and its phylogeny

**DOI:** 10.1080/23802359.2018.1532351

**Published:** 2018-10-26

**Authors:** Min-Jie Wu, Li-Li Yu

**Affiliations:** Shaoxing People’s Hospital, Shaoxing, PR China

**Keywords:** *Epeorus herklotsi*, Ephemeroptera, Mitochondrialgenome, phylogeny

## Abstract

The mitochondrial genome of *Epeorus herklotsi* (Ephemeroptera: Heptageniidae) from Longquan, Zhejiang province, China was a circular molecule of 15,499 bp in length, containing 13 protein-coding genes, two ribosomal RNAs, 23 transfer RNAs, and a control region. The A + T content of the overall base composition of H-strand is 65.7%. All of the 13 protein-coding genes were begun with ATN as start codon except ND5 using GTG. *ATP6*, *ATP8*, *COI*, *COIII*, *ND1*, *ND2*, *ND4L*, and *ND6* genes were terminated with TAA as stop codon, Cyt *b* ended with TAG and the other three protein-coding genes end with an incomplete stop codon (TA or T). In BI phylogenetic trees, the monophyly of the families Caenidae, Heptageniidae, Isonychiidae and Viemamellidae, and the genus *Epeorus* were strongly supported. *Epeorus* is a sister clade to *Parafronurus*, then cluster with *Paegniodes*.

The heptageniid mayflies *Epeorus herklotsi* is widely distributed throughout the rivers of China. For the convenience of identifying this species, we sequenced the mitochondrial genome of *E. herklotsi*. In Ephemeroptera, gene rearrangements were found in six sequences of twenty-three known mayfly mitochondrial genomes with IMQM tRNA cluster found in Heptageniidae (Zhang et al. 2008; Li et al. 2014; Tang et al. 2014; Gao et al. 2018). The phylogenetic relationships of Ephemeroptera still exist disputes in morphological and molecular methods (Kristensen 1981; Ogden and Whiting 2003; Zhang et al. 2008; Simon and Hadrys 2013; Li et al. 2014; Misof et al. 2014; Cai et al. 2018; Ye et al. 2018). Hence, we used the mitochondrial genome of *E. herklotsi* to analyse the characteristics of mitochondrial gene arrangement and to discuss the phylogenetic relationships within Ephemeroptera.

The samples of *E. herklotsi* from Longquan city, Zhejiang province, China were stored at −70 °C in our laboratory at Shaoxing People’s hospital. Some DNA fragments were amplified using highly conserved primers (Zhang et al. 2008). After obtaining most part of the mitogenome, we designed species-specific primers for the remaining part with reference to previously determined sequences. All polymerase chain reactions (PCRs) were carried out using a MyCyclere Thermal Cycler (Bio-Rad, Hercules, CA). *TaKaRa Ex-Taq* and *LA-Taq Kits* (Takara Biomedical, Dalian, China) were used for normal and long PCRs.

The complete mtDNA is 15,499 bp in length and contains 13 protein-coding genes, two ribosomal RNAs, 23 transfer RNAs genes, and noncoding regions. The overall base composition of H-strand is as follows: T (33%), C (21.3%), A (32.7%), G (13%), and the A + T content (65.7%). All of the 13 protein-coding genes were begun with ATN as start codon except ND5 using GTG. *ATP6*, *ATP8*, *COI*, *COIII*, *ND1*, *ND2*, *ND4L*, *ND6* genes were terminated with TAA as stop codon, Cyt *b* ended with TAG and the other protein-coding genes ended with an incomplete stop codon (TA or T). An IMQM tRNA cluster at the upstream of *ND2* gene was found, which was consistent with *P. youi* (Zhang et al. 2008) and three species of *Epeorus* (Tang et al. 2014; Gao et al. 2018), while it was different to *Paegniodes cupulatus* with the typical IQM tRNA cluster (Zhou et al. 2016).

Bayesian inference (BI) tree was constructed using the 13 PCGs from 21 species using *S. chinensis* (Li et al. 2014) as outgroup (Figure 1). BI analysis was performed by MrBayes version 3.1.2 (Huelsenbeck and Ronquist 2001). In the results, the monophyly of the families Caenidae, Heptageniidae, Isonychiidae, Viemamellidae, and the genus *Epeorus* was strongly supported. Isonychiidae was the basal clade to Ephemeroptera excluding Siphluriscidae. The monophyly of the family Siphlonuridae was not supported in our results, because *Siphlonurus* sp. (KM244684) and *S. immanis* (FJ606783) were cluster with *Ameletus* sp. 1 (KM244682) and *Ephemera orientalis*, respectively. Within the Heptageniidae clade, *Epeorus* was a sister clade to *Parafronurus*, then (*Epeorus + Parafronurus*) cluster with *Paegniodes*. Two species of *Epeorus herklotsi* were cluster together.

**Figure 1.**
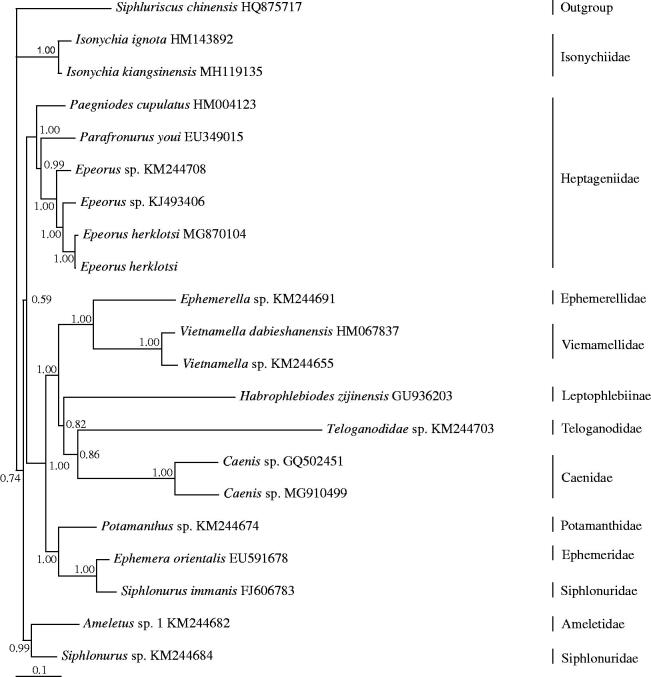
Phylogenetic tree of the relationships among 21 species of Ephemeroptera, including *Epeorus herklotsi* from Longquan, Zhejiang province, China based on the nucleotide dataset of the 13 mitochondrial protein-coding genes. The Bayesian posterior probability values are indicated above nodes. The GenBank numbers of all species are shown in the figure.

## Nucleotide sequence accession number

The complete mitochondrial genome of *E. herklotsi* has been assigned deposited to GenBank with the following accession number MH752075.
